# Risk of pain in the neck and shoulders and job change among hairdressers: a combined questionnaire and register-based Danish prospective cohort study

**DOI:** 10.1007/s00420-021-01753-4

**Published:** 2021-08-18

**Authors:** Jonathan Aavang Petersen, Charlotte Brauer, Lau Caspar Thygesen, Esben Meulengracht Flachs, Christina Bach Lund, Jane Froelund Thomsen

**Affiliations:** 1 Department of Occupational and Environmental Medicine, Bispebjerg and Frederiksberg Hospital, 2400 Copenhagen, Denmark; 2 National Institute of Public Health, University of Southern Denmark, Copenhagen, Denmark

**Keywords:** Musculoskeletal pain, Cohort studies, Epidemiology, Occupation, Hairdressers

## Abstract

**Objective:**

To investigate whether intensity of work as a hairdresser was associated with treatments for pain, and if musculoskeletal pain was associated with leaving the hairdressing trade.

**Methods:**

We formed two cohorts of hairdressers covered in the PensionDanmark Health Scheme (PDHS). Cohort 1 consisted of 1304 active hairdressers in 2009. Self-reported weekly haircuts were used as work intensity measure and treatments for pain in the neck and shoulders in PDHS were used as outcome. We used a Cox regression model with robust sandwich estimates adjusted for age, sex, employment status and prior treatment < 1 year before July 2009. Cohort 2 consisted of all hairdressers ever covered in the PDHS from 2006 to 2016 (*n* = 11,162). Exposure were treatments in PDHS within the last year. Outcome was leaving the trade within the following year. Adjustments were made for sex, calendar-year and age in Cox regression models.

**Results:**

The adjusted hazard ratio of treatments in PDHS compared to the lowest work intensity was 0.95 (95% CI 0.58–1.55) and 0.74 (0.43–1.29) for medium and highest intensity, respectively. The risk of leaving the trade was lower, HR 0.80 (0.72–0.90) among hairdressers with treatments in PDHS within the last year, mainly driven by hairdressers aged < 56 years.

**Conclusion:**

We found no association between intensity of work as a hairdresser, measured as self-reported weekly haircuts, and treatments for pain in PDHS. Furthermore, we found a protective effect of treatments in the PDHS within the last year on risk of leaving the trade.

## Introduction

Pain in the neck and upper extremities is highly prevalent among workers and in the general population, and is an important factor in change of occupation and early retirement (Côté et al. 2008; Hogg-Johnson et al. 2009; Hoy et al. 2014; Bevan 2015; Flachs et al. 2015). Besides requiring societal resources, personal losses can be involved both economically and career-wise (Woolf et al. 2012; Bevan 2015). Some evidence exists for an increased risk of diseases of the hands, elbows and shoulders for repetitive and forceful work (Thomsen et al. 2007; Van Rijn et al. 2010; Seidel et al. 2019). Static work, especially in combination with high repetition across the shoulder joint, may cause chronic pain in the neck and shoulder, but this is still not very well documented (Palmer and Smedley 2007; Côté et al. 2008; Mayer et al. 2012).

Hairdressers have physical work characterized by periods of high repetition and arm elevation, and low levels of muscular rest (Veiersted et al. 2008; Wahlström et al. 2009; Chen et al. 2010; Hanvold et al. 2015). At the same time, hairdressers report high levels of pain in the neck and upper extremities, with higher prevalence of neck and shoulder pain compared to non-hairdressing controls (Kozak et al. 2019; Mishra 2021). In a large study of more than 5000 hairdressers, 42 percent of those who had left the profession attributed pain in upper extremities as the main reason (Lysdal et al. 2011). Also, in a retrospective Finnish study of self-reported, health-related reasons for leaving the profession among hairdressers, an increased risk of 1.7 (95% CI 1.2–2.5) and 2.7 (95% CI 1.1–6.3) was found for neck/shoulder and hand/elbow disorders, respectively, compared to a control group of financial sector employees (Leino et al. 1999). In addition, a healthy survivor effect has been found among the hairdressing apprentices, where 1 in 5 leave the trade within 3 years and musculoskeletal pain and skin diseases were both reported by 47.4% as causes of leaving the trade (Foss-Skiftesvik et al. 2017).

The potentially harmful tasks and aspects of a hairdresser include styling and blow-drying where arms are elevated and the use of some force is required, and cutting hair which requires precise grasps on scissors and combs (Kozak et al. 2019). In addition, scarcity of breaks and high number of customers may further increase the risk of pain (De Smet et al. 2009; Nordander et al. 2016). However, intervention studies on healthy hairdressers addressing information on technique to reduce neck and shoulder workload or education programmes of young trainees have failed to significantly reduce complaints of pain (Crippa et al. 2007; Veiersted et al. 2008). Recently, the use of a new blow-dryer design was able to reduce time with upper-arm inclination angle above 60° and trapezius muscle activity during blow-drying. The participating hairdressers, however, preferred the traditional design (Wærsted et al. 2019).

Previously, investigation into causes of pain in the neck and upper extremities has been difficult due to their fluctuating nature. Epidemiological studies using register data based on hospital or health service databases may not find all cases. Using a health scheme, where access to treatment is easily available and not dependent on a doctor’s referral, as outcome source may reveal more cases. Self-reporting of both exposure and outcome entails risk of systematic recall bias influenced by current symptoms especially in cross-sectional designed studies (Gummesson et al. 2006; Miranda et al. 2006). Therefore, we designed two prospective studies with a low risk of recall bias.

In this study, we hypothesized, that (1) intensity of work as a hairdresser, defined objectively, was associated with treatments for shoulder and neck pain, and (2) musculoskeletal pain, defined objectively, was associated with leaving the hairdressing trade.

## Methods

### Study populations

The study was based on two prospective cohorts of hairdressers covered in the PensionDenmark Health Scheme (PDHS). To investigate hypothesis 1, the study population consisted of trained and currently active hairdressers responding to a questionnaire in 2009 with the aim of estimating prevalence of hand eczema and career consequences hereof, and has been described previously (Lysdal et al. 2011). Briefly, 7840 registered graduates from hairdressing vocational schools in Denmark from 1985 to 2007 received a postal questionnaire and 5239 respondents provided information on current occupational status. The questionnaire had 147 questions regarding various occupational and personal exposures. The characteristics of the cohort have been described previously, and consisted of 95.7% females with a mean working experience of 11.3 years (Lysdal et al. 2012). We identified the hairdressers describing themselves as active (*n* = 2863) who were covered in the PDHS after 1 July 2009, in the period following the collection of the questionnaire (*n* = 1304, cohort 1) using the personal identification number from the Danish Civil Registration System (Pedersen 2011).

Hypothesis 2 was investigated in a cohort of all persons ever covered in the PDHS as a hairdresser in the period 1st of January 2006 to 31st of December 2016 (*n* = 11,162, cohort 2). The definition as hairdresser was based on PDHS own classification of the members based on specific collective agreements.

## Variables

### Treatments in the PDHS

The PDHS provided members with access to medical services including physiotherapy, chiropractors, training and massage at multiple offsite private health clinics which has been described earlier (Pedersen and Arendt 2014). Members were mainly employees in manual work, skilled and unskilled. A not-for-profit labour market pension fund owned the PDHS. The PDHS has existed since 2005 with stepwise inclusion of jobs, among which the hairdressers were one of the first, enrolled in late 2005. Membership was provided by collective agreements between companies and workers, and the funding for the PDHS was agreed upon through collective agreements between unions of employees and trade organizations. The annual premium was 48 Euros per member and mandatory, tax-deductible and automatically deducted in the pension contributions. The use of the service was not reported to neither employer nor labour union and the number of treatments received was typically not restricted. No physician referral was required. The purpose of the PDHS was to prevent and treat work-related illness but injuries originating from leisure time activities have since been included. All individual treatments in the PDHS were registered at every visit. Type of treatment and body-region of concern were registered by the health care provider to the PDHS. In hypothesis 1, we used treatments for shoulder pain, neck pain or combinations of shoulder and neck pain as outcome, as these are among the most common complaint among hairdressers (Kozak et al. 2019). They were also chosen as exposure to high levels of repetition, arm elevation and low levels of muscular rest has been associated with neck and shoulder pain previously (Thomsen et al. 2007; Palmer and Smedley 2007; Côté et al. 2008; Van Rijn et al. 2010; Mayer et al. 2012; Seidel et al. 2019). All treatments (physiotherapeutic or chiropractic) within the last year were used as exposure in hypothesis 2.

### Intensity of work as a hairdresser (cohort 1)

From the questionnaire in 2009, a question regarding number of cuts in wet hair during the last working week was used as a proxy for customers/week (range 0–99) which was assumed to be the single most important ergonomic factor, as customer-handling tasks have been found to be the most strenuous task among hairdressers (Wahlström et al. 2009). Several other specific hairdressing tasks were also available from the questionnaire, but division into tertiles of exposures revealed a close correlation between number of cuts in wet hair and the other items. Therefore, we used number of cuts in wet hair as main exposure measure.

### Leaving the trade (cohort 2)

Individual information on job title as a hairdresser and working in the hairdressing industry was available in the Danish Occupational Cohort database (DOC*X) at Statistics Denmark which covers all work active residents in Denmark from 1970 using the International Standard Classification of Occupation (DISCO-88) and the Danish Industrial Classification of All Economic Activities (DB07) (Flachs et al. 2019). The hairdressers in cohort 2 were followed in the PDHS from date of entry until the last day of coverage. An individual was defined as having left the hairdressing trade if coverage ended and job title did not include “5141” (Hairdressers, barbers, beauticians and related workers) or industry code “960210” (Hair Salons) in the following year. However, we allowed return to the PDHS within 180 days, if coverage was as a hairdresser, without registering it as an event. Furthermore, we utilized the DREAM database where all persons who have received social benefits or any other transfer income since July 1991 are registered on a weekly basis (Hjollund et al. 2007). A person is considered to be self-supporting and consequently at work if no transfer payment is registered. We sub-divided those who left the hairdressing trade into five categories based on the DREAM register 1 year after the date of end of coverage: (1) In other work, (2) retirement pension, dead or emigrated, (3) early retirement, (4) long-term sickness absence (LTSA) or (5) education or unemployed. To define LTSA, we had to consider changing legislation regarding the period, where after the employers are entitled to reimbursement from the municipality corresponding to the sickness benefits. This period increased from 2 weeks until 2007 to 3 weeks in 2008 and 1 month in 2012. We defined LTSA as 8 or more consecutive weeks on sickness benefits.

### Statistical analysis

#### Hypothesis 1

Cohort 1 was followed from 1st July 2009 in the PDHS until the last day of coverage or 31st December 2018, whichever came first. A person was allowed as many periods of coverage as possible (e.g. if a person left the PDHS and returned at a later date as a hairdresser, the person was followed again). We used treatments for pain in the neck and shoulder as the outcome variable.

The main exposure of interest included the time-fixed variable number of cuts in wet hair divided into low, medium and high based on tertiles of exposure. We used Cox regression model with robust sandwich estimator to account for repeated events (treatments). Age was used as underlying risk time. We developed a directed acyclic graph (DAG) to determine which confounders to include in our final model containing age, sex, employment status (owner or employee) and prior treatment < 1 year before July 2009. From the questionnaire, we obtained information on smoking, employment status, height and weight and household income. Body mass index (BMI) was calculated as weight (kg)/height (m)^2^. Considering leaving the hairdressing trade was dichotomized to no and yes (within the near future, within 5 years/6–10 years and later). Years in the hairdressing trade was available in the questionnaire data using the Labour Market Supplementary Pension Scheme (Lysdal et al. 2011). We also included information on whether persons worked at least 32 h per week or worked part-time from the Danish Register-based Labour Force Statistics in 2009 (Petersson et al. 2011). Risk time in person-years was calculated as total number of days covered in the PDHS divided by 365.25. As sensitivity analyses, we adjusted for seniority in 2009 and working full-time or part-time and we excluded those responding 0 in exposure variable and excluded men and those with a prior treatment < 1 year before July 2009. Also, to address the issue of reverse causation, we restricted analyses to events within 1 year, and events after 1, 2 and 3 years of follow-up.

#### Hypothesis 2

Cohort 2 was followed from 1st January 2006 or date of entry in the PDHS until the last day of coverage or 31st December 2016, whichever came first. As exposure, we used any treatment in PDHS within the last year. Outcome was end of coverage in combination with not working as a hairdresser the following year. We also investigated the association with specific employment status defined by DREAM-status. Adjustments were made for sex and calendar-year. Age was used as underlying time in Cox regression models.

As sensitivity analyses, the effects in different age groups were investigated by restricting analyses to 18–55-year-olds and more than 55 years old. Also, as we might expect systematic different health behaviour among persons using the PDHS and those who did not, we restricted the cohort to only include those who had had any treatment. We also formed a cohort of hairdressers graduated in 2005 or later to restrict analyses to persons of similar seniority.

Data were analysed using SAS version 9.4 (SAS Institute Inc., Cary, NC, USA). Cox regression model was carried out using Proc PHREG in SAS.

## Results

### Baseline demographics

Cohort 1: Out of 2863 still active hairdressers in 2009, a total of 1304 was covered in the PDHS 1st of July 2009 and could be followed onwards. Of these, 1046 answered the question of work intensity and formed the cohort of hypothesis 1. The non-respondents of this item were quite similar to respondents on most characteristics, such as age, BMI, previous treatments, employment and smoking status, household income and considering leaving the trade, although they were slightly shorter and comprised fewer men (results not shown). Of the 1559 active respondents not included in this study, 91.2% were salon-owners. Also, they were older (mean age 38.1), had shorter seniority (mean 9.88 years), were less prone to considering leaving the trade (30.1%) and had a slightly higher income than the included hairdressers. Similar distribution of smoking status and work intensity was found.

Divided into tertiles of work intensity, the high work intensity group was slightly, but significantly older, more likely to smoke than low- and medium-exposed groups and more likely to work full-time. Also, the composition of sex in the three groups varied significantly, with more men in the higher exposed group (Table 1). Seniority was closely correlated to age at entry (Spearman correlation coefficient = 0.66 *P* < 0.0001) and low-exposed had significantly fewer years in the hairdressing trade. Using number of cuts in wet hair as a continuous variable, this corresponded to 2.7 additional cuts per week per 10 years increase in seniority. The exposure groups were quite similar regarding other characteristics, see Table 1.

**Table 1:** Characteristics of the cohort of hairdressers responding a questionnaire in 2009, covered in the PensionDanmark Health Scheme in the period 1st July 2009–1st January 2019 (cohort 1)

Work intensity (*n* cuts in wet hair/week in tertiles)	Low 0–27 cuts	Medium 28–40 cuts	High 41–99 cuts	*P* value for difference	Total
Total, *n* (RT)	355 (2048)	380 (2293)	311 (1928)		1046 (6269)
Men, *n* (RT)	7 (39)	10 (49)	16 (97)		33 (185)
Women, *n* (RT)	348 (2009)	370 (2243)	295 (1830)	**0.0499**^b^	1013 (6083)
Mean age at start of follow-up (IQR)	34.5 (30.3–38.2)	35.6 (31.0–40.5)	35.5 (30.9–40.1)	**0.0148**^c^	35.2 (30.8–39.6)
Mean seniority in 2009, years (IQR)^e^	12.0 (8–15)	13.5 (9–18)	13.3 (9–17)	**0.0006**^c^	12.9 (9–17)
BMI (1034 responded) (IQR)	23.3 (20.8–25.0)	23.3 (20.8–25.4)	23.8 (20.8–26.3)	0.1183^c^	23.5 (20.8–25.4)
Height, m (1041 responded) (IQR)	1.69 (1.64–1.72)	1.68 (1.64–1.72)	1.68 (1.64–1.72)	0.5058^c^	1.68 (1.64–1.72)
Prior treatment < 1 year before July 2009, *n* (%)^a^	22 (6.2)	32 (8.4)	16 (5.1)	0.2069^b^	70 (6.7)
Employment status (1037 responded)					
Owner, n ( %)	20 (5.7)	24 (6.4)	16 (5.2)		60 (5.8)
Employee, n ( %)	331 (94.3)	352 (93.6)	294 (94.8)	0.7895^b^	977 (94.2)
Working hours per week in 2009^d^					
≥ 32 h, n (%)	164 (46.2)	184 (48.4)	178 (57.2)		50.3
< 32 h, n (%)	156 (43.9)	156 (41.1)	112 (36.0)		40.5
Unregistered, n (%)	35 (9.9)	40 (10.5)	21 (6.8)	**0.0410**^b^	9.2
Household income (822 responded)					
Below 299,000, n (%)	35 (12.6)	43 (14.3)	48 (19.8)		126 (15.4)
300,000–549,000, n ( %)	83 (30.0)	87 (28.9)	69 (28.4)		239 (29.1)
550,000–749,000, n (%)	119 (43.0)	119 (39.5)	96 (39.5)		334 (40.6)
750,000 and above, n (%)	41 (14.4)	52 (17.3)	30 (12.4)	0.2802^b^	123 (15.0)
Smoking (1040 responded)					
Yes (incl. sometimes), n (%)	108 (30.6)	139 (36.8)	123 (39.8)		370 (35.6)
No, n (%)	245 (69.4)	239 (63.2)	186 (60.2)	**0.0394**^b^	670 (64.4)
Considering leaving the profession (1036 responded)					
No, n (%)	169 (48.4)	203 (53.9)	155 (50.0)		527 (50.9)
Yes, n (%)	180 (51.6)	174 (46.1)	155 (50.0)	0.3222^b^	509 (49.1)

Estimates in bold significant at *P* < 0.05

*RT* risk time in person-years, *BMI* body mass index, *IQR* interquartile range

^a^Prior treatment for pain in the shoulders and/or neck

^b^Chi-square/Fisher’s exact test

^c^One-sided ANOVA

^d^According to the Register-based labour force statistics at Statistics Denmark

^e^Based on the annual affiliation to the hairdressing trade from the Labour Market Supplementary Pension Scheme

Cohort 2: A total of 11,162 hairdressers covered in the PDHS were followed for 41,459 person-years. The respondents of the questionnaire had a slightly higher age of entry to the PDHS and consisted of fewer male hairdressers, but the distribution of treated body region was similar, dominated by neck, shoulders and back (Table 2).

**Table 2:** Characteristics of cohort 1 and 2

	Cohort 1		Cohort 2	
Number (person-years)	1304	(7772)	11,162	(41,459)
Females, %	97%		94%	
Age of entry to the PDHS, mean (IQR)	31.9	(27.4–36.4)	29.1	(22.0–33.5)
Grouped age of entry to the PDHS, *n* (%)				
18–30	555	(43)	7456	(67)
31–40	619	(47)	2139	(19)
41–50	127	(10)	969	(9)
51–60	3	(0.2)	459	(4)
61–70	0	(0)	139	(1)
Treated body region, *n* (%)				
Arm	32	(0.6)	145	(0.7)
Elbow	69	(1.2)	263	(1.3)
Wrist/hand	21	(0.4)	134	(0.7)
Shoulder	325	(5.7)	971	(4.8)
Neck/shoulder	282	(4.9)	1231	(6.0)
Neck	3226	(56.2)	10,876	(53.2)
Back	1077	(18.8)	4348	(21.3)
Leg	87	(1.5)	342	(1.7)
Other	188	(3.3)	887	(4.3)
N/A	430	(7.5)	1266	(6.2)
Total treatments	5737		20,463	
Not hairdresser 1 year after leaving PDHS (persons^a^)		–	5146	(4564)
Specific employment status, *n* (%)				
In other work		–	3706	(72.0)
Retirement pension/dead/left the country		–	270	(5.3)
Early retirement		–	47	(0.9)
LTSA etc		–	445	(8.7)
Education/ unemployed		–	678	(13.2)

*PDHS* PensionDanmark Health Scheme, *IQR* interquartile range, *N/A* no body region assigned, *LTSA* long-term sickness absence (≥ 8 weeks)

^a^Persons with one or more events

### Risk factors for neck and shoulder treatments in the PDHS (hypothesis 1)

A total of 229 of 1046 hairdressers (21.9%) were treated 3101 times for neck and shoulder pain by the physiotherapists or chiropractors with a median of 6.0 treatments per person and a range of 1–100 treatments. In univariate analyses, considering leaving the profession in 2009 and having previous treatments before start of follow-up were significant risk factors for neck and shoulder treatments in PDHS (results not shown). Being the owner of the workplace was a significant protective factor compared to employees.

Using number of cuts in wet hair per week divided in tertiles as work intensity, the incidence rates (IR) of treatments in low, medium and high were 53.6, 52.4 and 41.5 per 1000 person-years, respectively (Table 3). After adjustments for sex, prior treatment < 1 year before July 2009 and employment status, we found no significant difference in hazard ratio (HR) between low-, medium-, and high-exposure groups. However, a tendency towards an inverse relationship between intensity and outcome was found. Sensitivity analyses excluding those responding 0 in exposure, excluding men and those with a prior treatment < 1 year before July 2009 or restricting analyses to events within 1 year and events after 1, 2 and 3 years of follow-up did not change the results. Including seniority in 2009 or working full-time or part-time as a covariate did not change the results.

**Table 3:** Associations between repeated treatments for pain in the neck/shoulder and work intensity per week, incidence rate per 1000 person-years (IR), crude and adjusted hazard ratio (HR) among covered hairdressers (cohort 1)

Cuts in wet hair/week	Low 0–27 cuts	Medium 28–40 cuts	High 41–99 cuts	Total
Number of treatments	1098	1202	801	3101
Person years	2048	2293	1928	6269
IR	53.6	52.4	41.5	49.5
HR, crude	1	0.95 (0.58–1.55)	0.76 (0.44–1.31)	
HR, model 1^a^	1	0.95 (0.58–1.55)	0.74 (0.43–1.29)	
HR, model 2^b^	1	0.88 (0.55–1.42)	0.79 (0.46–1.37)	
HR, model 3^c^	1	0.90 (0.56–1.45)	0.79 (0.46–1.36)	

Numbers in brackets are 95% Confidence Intervals. Estimates in bold significant at *P* < 0.05

^a^Adjusted for sex

^b^Adjusted for sex and prior treatment < 1 year before July 2009

^c^Adjusted for sex, prior treatment < 1 year before July 2009 and employment status

### Treatments in PDHS and risk of leaving the trade (hypothesis 2)

Among the 11,162 hairdressers, 5146 events happened where a person left the PDHS and was not registered as working as a hairdresser in the following year (Table 2). The events were distributed among 4564 persons (40.9%), as we allowed the return to PDHS if the coverage was registered as a hairdresser. Seventy-two percent had found another job with the most common jobs being child-care worker, shop assistants, home-based personal care workers, secretaries and office workers. A total of 445 hairdressers had more than 8 weeks of sickness absence (LTSA) in the following 52 weeks after coverage ended in the PDHS. Only 47 was registered as being on early retirement after end of coverage.

The overall risk of leaving the hairdressing trade was significantly lower among hairdressers with treatments in PDHS within the last year with an adjusted HR of 0.80 (95% CI 0.72–0.90), see Table 4. Stratifying on age group, the effect was only significant among those aged 18–55 years, with an adjusted HR of 0.79 (95% CI 0.71–0.89). The same pattern was evident in the risk of leaving the hairdressing trade for another job, retirement pension and education or unemployment, whereas risk of early retirement did not reach statistical significance. However, treatments in PDHS within the last year increased risk of leaving the hairdressing trade and being on LTSA many-fold, although insignificantly.

**Table 4:** Association between having received treatments in the PensionDanmark Health Scheme within the last year and leaving the hairdressing trade, adjusted hazard ratio (HR (95%CI)) among covered hairdressers (cohort 2) and stratified by age

Treatments within 1 year	Yes
Left the hairdressing trade, (*n* = 5146)	350
HR, adjusted^a^	**0.80 **(**0.72–0.90**)
HR, adjusted^a^ 18–55 years	**0.79 **(**0.71–0.89**)
HR, adjusted^a^ above 55 years	0.88 (0.63–1.24)
Left the hairdressing trade for another job, (*n* = 3706)	254
HR, adjusted^a^	**0.83 **(**0.73–0.95**)
HR, adjusted^a^ 18–55 years	**0.82 **(**0.72–0.94**)
HR, adjusted^a^ above 55 years	0.98 (0.62–1.55)
Left the hairdressing trade for retirement pension, death or emigration, (*n* = 270)	16
HR, adjusted^a^	**0.59 **(**0.36–0.98**)
HR, adjusted^a^ 18–55 years	0.25 (0.06–1.03)
HR, adjusted^a^ above 55 years	0.75 (0.43–1.29)
Left the hairdressing trade for early retirement, (*n* = 47)	4
HR, adjusted^a^	0.70 (0.25–1.95)
Left the hairdressing trade for LTSA^b^, (*n* = 445)	51
HR, adjusted^a^	1.22 (0.94–1.70)
HR, adjusted^a^ 18–55 years	1.22 (0.91–1.65)
HR, adjusted^a^ above 55 years	7.80 (0.88–69.43)
Left the hairdressing trade for education/unemployment, (*n* = 678)	25
HR, adjusted^a^	**0.43 **(**0.29–0.64**)
HR, adjusted^a^ 18–55 years	**0.44 **(**0.30–0.66**)
HR, adjusted^a^ above 55 years	–

Estimates in bold significant at *P* < 0.05. Numbers in brackets are 95% Confidence intervals

^a^Adjusted for sex and calendar-year

^b^Long-term sickness absence (≥ 8 weeks)

To avoid the effect of a possible information/behaviour bias, we isolated hairdressers with any treatment in the PDHS (*n* = 1767) and repeated the analysis on the second and following treatments (results not shown). The pattern shifted towards an increased risk among those treated within the last year regarding leaving the hairdressing trade for all reasons, for another job and for early retirement. However, none reached statistical significance and confidence intervals were wider, due to smaller sample size.

As a further sensitivity analysis, we created a sub-cohort of trained hairdressers graduated in 2005 or later constituting 3716 persons. Among these, 1519 (41%) left the trade during the follow-up. The same pattern of a protective effect of having a treatment in the PDHS on leaving the trade was found, with an HR of 0.72 (95% CI 0.59–0.89).

## Discussion

### Key results

The purpose of this study was to investigate, in a prospective design using objectively defined exposure and outcome, whether intensity of work as a hairdresser was associated with treatments for shoulder and neck pain in the PDHS, and if musculoskeletal pain, defined as registered physiotherapeutic or chiropractic treatments, was associated with leaving the hairdressing trade.

We did not find a significant association between tertiles of self-reported intensity of work defined as reported number of cuts in wet hair per week in 2009 and later treatments for shoulder and neck pain. Furthermore, we found a protective effect of having treatments in the PDHS within the last year on risk of leaving the trade for another job and for education or unemployment, but only statistically significant among hairdressers aged 18–55 years. However, an increased risk of leaving the trade and being on LTSA was found, although insignificant.

### Strengths and limitations

The strengths of this study were the prospective design and the homogeneous study population with only a few characteristics distributed unevenly among the selected exposure groups. Also, the use of registered treatments as an objective indicator for pain eliminates the effects of recall bias. The use of the DREAM database has the advantage of avoiding bias due to self-reports and enables a full follow-up on all persons in the cohort. The DOC*X-database has been validated with substantial agreement between DISCO-88 codes assigned from self-reported job titles and DISCO-88 codes registered in the DOC*X (Petersen et al. 2019). This enables the coupling of the PDHS and the DOC*X register data and ensures a high level of confidence in the job titles used as outcome for leaving the trade.

One limitation of this study could be the choice of exposure variable in hypothesis 1. This was based on a single item in the questionnaire (number of cuts in wet hair per week) in the respondents’ last working week. A few participants answered zero or a very low number, which is not in accordance with the typical work as a hairdresser. This may have caused a non-differential misclassification of exposure and may have attenuated results. However, the average number of cuts in wet hair per week was similar to the mean number of customers in other studies on hairdressers (Veiersted et al. 2008; Chen et al. 2010). Furthermore, the exposure variable was not differentiated by women’s or men’s specialty, where some evidence suggested different exposure to use of force, longer average time to finish a woman’s haircut and higher overall wrist velocity among female hairdressers (Chen et al. 2010). However, we have no knowledge of the sex-specific specialisation among Danish hairdressers, and we expect this to be minor. The self-reported exposure as a time-fixed variable might cause misclassification, if in fact, the average number of customers varies significantly throughout the follow-up. However, sensitivity analyses restricting on events after 1, 2 and 3 years of follow-up did not change the results, and we therefore consider the exposure variable to be valid. Further studies are needed with exposure measurements spanning longer periods of time.

Another limitation may be the novel use of PDHS as a measure for incidence of pain. We do not know if there is some unmeasured confounding of the use of the PDHS among the hairdressers. This might lead to skewed results due to different levels of information about the possibility of treatment or different health behaviour among the hairdressers. The concern that some workers may be unaware of their access to the PDHS has been raised previously by Pedersen and Arendt, who found no indication of a decrease in the physician-prescribed physical therapy among plumbers after the introduction into the PDHS, but found a small decrease among electricians (2014). The PDHS is, however, designed to be freely available and allows for easy and unrestricted access to treatments. The hairdressers were among the very first occupational groups included in the PDHS in 2005, and we therefore do not expect the use of treatments in the PDHS after 2009 to be biased. This is supported by the sensitivity analysis among the hairdressers who ever used the PDHS.

Psychosocial factors have been associated with MSDs among hairdressers (Kozak et al. 2019). Psychosocial factors, such as mental stress, lack of acknowledgment or motivation to continue, may have influenced our results, but we had only information on psychosocial factors on hairdressers considering leaving the hairdressing trade. We therefore did not include this in the models.

### Interpretation

The pattern of an apparent protective effect of increasing intensity of work may be caused by a healthy worker effect among hairdressers of different seniority at inception into the cohort. The healthy worker effect is due to the self-selection by individuals capable of performing these tasks, which in turn may be related to the observed use of the PDHS, leading to possible erroneous conclusions (Chowdhury et al. 2019). However, the healthy worker effect is expected to be greater when comparing the worker’s outcomes with a general population and to a lesser degree when the workers are divided into groups as per their exposure status as we did (Chowdhury et al. 2019). We did find a slight but significantly higher seniority with increasing work intensity. We did not include seniority in the main models as it was closely correlated to age, which was used as underlying risk time. Sensitivity analysis including seniority did not change the results.

The main treated anatomical areas among the hairdressers were neck, shoulders and back, a distribution like the findings of other studies on hairdressers, primarily based on self-reports (Kozak et al. 2019). Pain in the neck and shoulders have been associated with working with elevated arms, repetitive movements of the arms, high use of force and hand-arm vibration (Svendsen et al. 2013; Linaker and Walker-Bone 2015). Hairdressers are typically exposed to high levels of arm elevation, repetitive movements and some tasks with some use of force (Wahlström et al. 2009; Nordander et al. 2016). One prospective study, using repeated self-reported exposures among hairdressers, has found a small but significant association between workload and incident self-reported neck and shoulder pain (Hanvold et al. 2014). Arm elevation was found to be associated with shoulder pain in a study on hairdressers; however, sample size and low levels of reported pain make comparisons with our study difficult (Hanvold et al. 2015).

An important risk factor for pain in the neck and shoulders is the psycho-social work environment (Kraatz et al. 2013; Kim et al. 2018). We did not have information on psycho-social risk factors in cohort 2 and in the questionnaire in cohort 1, psycho-social risk factors were restricted to those considering leaving the trade reducing statistical power, and risking biased results. Among 165 hairdressers with a mean seniority much like in this study (12.3 years SD 10.8), self-reported psychosocial factors, such as excessive work, burnout and emotional exhaustion, were risk factors for self-reported work-related upper limb disorders (De Smet et al. 2009). The cross-sectional design did not allow for causal inferences and the self-reported exposure and outcome may have caused some recall bias.

A questionnaire study in France reported a higher prevalence of musculoskeletal injuries among the self-employed compared to wage-earning hairdressers (Deschamps et al. 2014). This was based on self-reported outcomes and our study found a lower risk among owners vs employees when using treatments in the PDHS. However, our cohort mainly consisted of employed hairdressers (94.2%), as the PDHS mainly covers those working under collective agreements, and this may have influenced the results.

Our study found a high frequency of career change among hairdressers covered in the PDHS between 2006 and 2016, where 4564 out of 11,162 (40.9%) no longer worked in the trade 1 year after leaving the PDHS. Among the 5146 events, 72% were registered as working in a different trade without the use of social benefits and 13.2% were under further education or on unemployment benefits. The same was found among a sub-cohort of trained hairdressers graduated in 2005 or later, where 25% had left the trade, primarily for another job, within the ten-year follow-up period. This is comparable to the 21.8% who had left the trade out of 248 hairdressing apprentices in Denmark during 3 years of follow-up, where the main self-reported reason for leaving was musculoskeletal pain and skin diseases (Foss-Skiftesvik et al. 2017). Lysdal et al. reported an average of 8.4 years of work as an hairdresser before leaving the trade among 2321 ex-hairdressers (Lysdal et al. 2011). It is impossible to directly compare this with our findings due to design differences. However, our results also indicate a large turnover in the hairdressing trade. The use of the PDHS seems to protect against leaving the trade, in line with the intention of the PDHS to promote health and reduce consequences of injuries among workers. At the same time, we did find indications of a shift in risk direction when restricting the analysis to hairdressers in the cohort with at least one treatment. This was statistically insignificant but could indicate either an effect of severity of the treated conditions or an effect of knowledge about the health scheme. Also, the protective effect was only significant among younger hairdressers compared to those aged > 55 years and still strong among the sub-cohort of novice hairdressers. This might indicate a stronger response to early intervention or better prognosis among young hairdressers affected by pain-causing conditions. As we could not randomly assign treatments to the cohort, the causative effect is uncertain. Three years after the introduction of the PDHS in electricians and plumbers, Pedersen and Arendt found a small reduction in annual number of physician contacts and health-related job absenteeism (2014). This, in combination with our results, indicates a positive effect of the PDHS on some work-related outcomes. We do, however, need additional studies on other job groups, to conclude this.

## Conclusion

We did not find an association between number of haircuts per week, measured as a time-fixed exposure in 2009 and treatment of neck and shoulder pain within PDHS. This may be due to a healthy worker effect and/or the use of a time-varying exposure analysed as a time-fixed exposure. Furthermore, we found a protective effect of the use of the PDHS on the risk of leaving the trade as a hairdresser. However, an increased risk of leaving the trade and being on LTSA was found. The study warrants further investigation of the use of PDHS in other job groups and prospective studies with individual and repeated measurements of exposure.

## Data Availability

Data from the Danish national registers are available through online access at Statistics Denmark under standard conditions. Data from the Lysdal questionnaire are available from the Danish National Archives under standard conditions. Data from PensionDanmark are not available.
